# A Large Cohort Study of Hypothyroidism and Hyperthyroidism in Relation to Gynecologic Cancers

**DOI:** 10.1155/2013/743721

**Published:** 2013-07-15

**Authors:** Jae H. Kang, Angela S. Kueck, Richard Stevens, Gary Curhan, Immaculata De Vivo, Bernard Rosner, Erik Alexander, Shelley S. Tworoger

**Affiliations:** ^1^Channing Division of Network Medicine, Brigham & Women's Hospital, Harvard Medical School, Boston, MA, USA; ^2^Division of Gynecologic Oncology, University of Connecticut Health Center, 263 Farmington Avenue, MC1614, Farmington, CT 06034, USA; ^3^Division of Epidemiology & Biostatistics, Department of Community Medicine and Health Care, University of Connecticut Health Center, Farmington, CT, USA; ^4^Department of Epidemiology, Harvard School of Public Health, Boston, MA, USA; ^5^Department of Biostatistics, Harvard School of Public Health, Boston, MA, USA; ^6^Division of Endocrinology, Brigham & Women's Hospital, Harvard Medical School, Boston, MA, USA

## Abstract

*Background*. Thyroid status may influence tumorigenesis of gynecologic cancers, yet epidemiologic studies of this relationship are limited and inconsistent. *Methods*. We evaluated the association of self-reported history of physician-diagnosed hypothyroidism and hyperthyroidism with medical-record confirmed endometrial (EC; all invasive adenocarcinomas) and ovarian cancer (OC; epithelial ovarian or peritoneal cancers) in Nurses' Health Study (NHS) from 1976 to 2010 and NHSII from 1989 to 2011. Cox proportional hazard models were used to estimate multivariable rate ratios (RRs) and 95% confidence intervals based on pooled cohort data. *Results*. We confirmed 1314 incident cases of EC and 1150 cases of OC. Neither a history of hypothyroidism nor hyperthyroidism was significantly associated with risk of EC or OC. However, having a history of hypothyroidism for 8+ years (median) was nonsignificantly inversely associated with EC (RR = 0.81; 95% CI = 0.63–1.04; *P*-trend with history duration = 0.11) and OC (RR = 0.87, 95% CI = 0.66–1.15; *P*-trend = 0.13). Having a history of hyperthyroidism for 6+ years (median) was non-significantly positively associated with EC (RR = 1.69; 95% CI = 0.86–3.30; *P*-trend = 0.12) but not OC (RR = 1.12; 95% CI = 0.46–2.72; *P*-trend = 0.95). *Conclusions*. A history of hypothyroidism or hyperthyroidism was not significantly associated with risk of EC or OC.

## 1. Introduction

Endometrial cancer (EC) is the most common gynecologic malignancy in the United States, and the fourth most common cancer overall in women [1]. Most cases are diagnosed in postmenopausal women at an early stage and have an excellent prognosis; however, those presenting with advanced or recurrent disease have limited treatment options and survival of less than 3 years [2, 3]. The number of deaths from EC has doubled over the last 20 years, and the reason for this is not well understood [4]. Well established risk factors for EC are hormonally linked and include a greater lifetime exposure to estrogen, obesity, and diabetes and display a progression from atypical hyperplasia to cancer [5, 6]. Similarly, ovarian cancer (OC) risk is estrogen-linked but very little is known about the etiology or its precursors and it remains the most lethal gynecologic cancer [7]. The hypothalamic-pituitary axis (HPA) regulates the female reproductive tract not only through the regulation of estrogen and other sex hormones but also through interactions with related pathways including thyroid hormones [8]. 

 Thyroid hormones (T3 and T4) are important regulators of differentiation, growth, and metabolism of virtually all tissues including the ovaries and endometrium [8–11]. Thyroid disorders can present as hyperthyroidism (excessive T3 and T4 production) or more commonly as hypothyroidism (decreased T3 and T4 production) with a higher prevalence of both disorders affecting women [12]. Menstrual irregularities, anovulation, and infertility are common symptoms of both types of thyroid disorders and result from both direct and indirect effects on the endometrium and ovaries [13, 14]. Direct binding of thyroid hormones to receptors on ovarian surface epithelium, ovarian follicles, and the endometrial lining influences proliferation and regulation of menstruation [15, 16]. Indirectly, alterations in thyroid hormone levels cause changes in serum levels of sex hormone binding globulin (SHBG), sex hormones (estrogen and testosterone), and their metabolism and clearance from the body [17, 18]. These alterations in hormone levels may then impact the risk of EC or OC.

Due to the ubiquitous role of thyroid hormones, it has been hypothesized that they may influence cancer development and growth in various tissues [19–21]. This association has recently been clarified after a membrane cell surface receptor for thyroid hormones was characterized on rapidly dividing cells (e.g., endometrium) in contrast to the usual intracellular transport and nuclear binding in other tissues [22, 23]. Several *in vitro* and *in vivo* studies have implicated thyroid hormones in tumorigenesis and cell proliferation in glioma, gastric, and breast cancer cell lines [21]. Blocking thyroid hormone receptors or decreasing T3 and T4 production to induce hypothyroidism has been shown to reduce tumor growth in breast, prostate and hepatocarcinoma mouse models [20–23]. 

Observational studies in lung, colon, prostate, and breast cancer patients have reported conflicting results regarding the relationship of thyroid hormones with increased cancer risk [24–27]. A recent large population study in Norway reported an association between hyperthyroidism and risk of lung and prostate cancer, but gynecologic cancers were not assessed [28]. Only one record-linkage analysis has examined the relationships of uterine and ovarian cancer risk with pre-existing chronic conditions and reported that thyroid diseases (types of disease were not specified) were associated with a significant risk of EC (RR 1.52) [29].

Given that thyroid disorders and alterations in thyroid hormone expression influence ovulation, endometrial physiology, and estrogen levels, further exploration into the possible association with gynecologic cancer risk is warranted. Thus, we evaluated the association between self-reported history of physician-diagnosed hypothyroidism and hyperthyroidism in Nurses' Health Study (NHS) from 1976 to 2010 and NHSII from 1989 to 2011 in relation to risk of medical-record confirmed ECs and OCs.

## 2. Methods

### 2.1. Study Population

The NHS started in 1976 with 121,700 US female registered nurses aged 30–55 years, and the NHSII started in 1989 with 116,430 U.S. female registered nurses aged 25–42 years. Participants completed baseline questionnaires in both cohorts and since have been followed with biennial questionnaires, providing information on various lifestyle factors and diagnosed diseases. Participant followup is high in both cohorts; >90% of the total possible person-years through June 2010 in the NHS and through June 2011 in the NHSII were obtained. This investigation was approved by the Institutional Review Board of the Brigham and Women's Hospital and/or the Harvard School of Public Health.

### 2.2. Ascertainment and Validation of Hypothyroidism and Hyperthyroidism

#### 2.2.1. Ascertainment of Hypothyroidism Diagnosis

*NHS*. On the 2002 questionnaire, participants were asked about ever having had a diagnosis of physician-diagnosed hypothyroidism and the date of diagnosis. In addition, participants had the opportunity to report physician-diagnosed hypothyroidism on each biennial questionnaire from 1978 to 2008, where they were asked to write-in physician-diagnosed diseases; write-in diseases assigned with ICD codes of “244” were considered self-reports of hypothyroidism. 

*NHSII.* On the baseline 1989 questionnaire, participants were asked about a “history of thyroid disease” diagnoses (without specifying hyperthyroidism or hypothyroidism), date of diagnosis, and current use of thyroid hormone replacement therapy. Those who reported having a “thyroid disease” diagnosis as well as reported currently using thyroid hormone replacement therapy were considered as having a history of hypothyroidism, as in our data from later cycles; among those who report thyroid hormone replacement therapy at a given cycle, a report of hypothyroidism was more than 10 times more common than a report of hyperthyroidism in the same cycle. In 1993, participants were asked if they had developed hypothyroidism and the date of diagnosis, and starting from 1995 to 2009, they were asked if they had developed hypothyroidism in the preceding 2 years. As in the NHS, from 1989 to 2009, participants also had the opportunity to write-in hypothyroidism; write-in reports assigned with ICD codes of “244” were considered as hypothyroidism reports. 

#### 2.2.2. Ascertainment of Hyperthyroidism Diagnosis

*NHS.* On the 2002 questionnaire, participants were asked about ever having had a diagnosis of physician-diagnosed hyperthyroidism (“Graves' disease/Hyperthyroidism”) and the date of diagnosis, and starting in 2004–2008, they were asked about hyperthyroidism diagnoses in the preceding 2 years. In addition, participants had the opportunity to report physician-diagnosed hyperthyroidism on each biennial questionnaire from 1978 to 2008, where they were asked to write-in physician-diagnosed diseases; write-in diseases assigned with ICD codes of “242” were considered self-reports of hyperthyroidism.

*NHSII.* In 1993, participants were asked if they had developed Graves' disease before or after baseline, and starting in 1995, they were asked if they had developed “Graves' disease” or “Graves' hyperthyroidism” in the preceding 2 years. As in the NHS, from 1989 to 2009, participants also had the opportunity to write-in hyperthyroidism diagnoses; write-in reports assigned with ICD codes of “242” were considered as hyperthyroidism reports. 

#### 2.2.3. Validation of Hypothyroidism and Hyperthyroidism

*Hypothyroidism.* We conducted a pilot study among NHSII participants who had self-reported hypothyroidism on biennial questionnaires. First, we sent participants a supplementary questionnaire asking about their diagnosis date, medication use, prior history of hyperthyroidism, postpartum thyroiditis, thyroid treatment (radioactive iodine treatment, thyroid surgery, etc.), and family history of hypothyroidism. In addition, we asked participants for permission to retrieve their medical records from diagnosing health care providers. We received relevant medical records for 68 participants; of these, a clinical diagnosis of hypothyroidism and current levothyroxine/desiccated thyroid prescription was documented in 100%, and in 56 (82%), laboratory results demonstrated abnormal and persistently elevated TSH values (>4.5 mIU/L) or presence of Hashimoto's thyroiditis based on clinical factors (family history) in addition to biochemical (TPO Ab > 20 IU/mL without TSH > 4.5 mIU/L) and/or radiologic markers (thyroid ultrasound with diffuse heterogeneous pattern). Thus, we can successfully identify cases of primary hypothyroidism in these populations of health care professionals.

*Hyperthyroidism. *A validation study was performed among 72 women in NHSII who reported having Graves' hyperthyroidism on the biennial questionnaire to assess the reliability of self-reports [30]. Supplemental questionnaires and requests for permission to obtain medical records from the treating physician were sent to these participants. The supplemental questionnaire asked about whether they had been diagnosed as having Graves' disease or Graves' hyperthyroidism and the year of diagnosis; symptoms and signs of hyperthyroidism; whether they had goiter or eye changes; tests done to confirm the diagnosis (thyroid function tests, thyroid scan and uptake, and ophthalmologic examination); antithyroid drug, radioiodine, or surgical treatment; and diagnosis of other thyroid disorders (hyperfunctioning nodule, multinodular goiter, hypothyroidism, or thyroid cancer). The diagnosing physicians of record were asked to send copies of medical information confirming the diagnosis, if participants granted permission. Criteria for the diagnosis of Graves' hyperthyroidism included test results indicative of hyperthyroidism, initiation of treatment for hyperthyroidism, and absence of other thyroid disorders. Based on review of the supplemental questionnaire and medical records, the diagnosis of Graves' hyperthyroidism was corroborated in 61 (85%) of the 72 women, demonstrating the high validity of the self-reports of Graves' hyperthyroidism in this population. 

### 2.3. Identification of Cancer Cases

In both NHS and NHSII, we collected information on new EC and OC diagnoses on each questionnaire at baseline and on all biennial questionnaires. In addition, deaths due to EC or OC were identified through information from family members, the U.S. National Death Index, or the U.S. Postal Service. For all reported cases and deaths, we obtained medical records related to the diagnosis. For both outcomes, a gynecologic pathologist masked to thyroid disease status reviewed the medical records to confirm the diagnosis, stage, histologic type/subtype, and degree of invasiveness. For EC, we identified 1314 cases of invasive adenocarcinomas defined by the International Federation of Gynecology and Obstetrics (FIGO) as stages IB to IVB diagnosed between June 1976 and June 2010 in NHS and June 1989 and June 2011 in NHSII. (In alternate analyses of 1786 cases of stage 1A to IVB, results were similar (data not shown); thus, these analyses focused on the invasive cancers of stages IB to IVB, which are of greater clinical and public health significance). For OC, we included all epithelial OC and primary peritoneal cases confirmed by pathology report review (*n* = 1150) diagnosed during the same period.

### 2.4. Statistical Analysis

Analyses were conducted separately for each outcome using the combined NHS and NHSII datasets. At baseline, we excluded women with existing cancer other than nonmelanoma skin cancer, women with missing data on the dates of diagnoses of hypothyroidism or hyperthyroidism and women reporting other thyroid disorders. Participants accrued person-time from the return date of the baseline questionnaire until the date of EC/OC diagnosis, diagnosis of any other cancer (excluding nonmelanoma skin cancer), diagnosis of other thyroid disorders, death, or end of followup. 

For EC, at baseline and during followup, we excluded women who reported a hysterectomy from contributing person-time. The total numbers of women who ever contributed person-time in the EC analyses were 95,135 in the NHS and 105,027 in the NHSII. For OC, at baseline and during followup, we censored women with bilateral oophorectomy and menopause due to pelvic irradiation. The total numbers of women who ever contributed person-time in the OC analyses were 108,609 in the NHS and 108,733 in the NHSII.

We used time-varying Cox proportional hazards regression models, stratified by age, time period at risk (from one questionnaire return to the return of the next questionnaire/end of followup), and cohort (NHS or NHSII) to model the age-adjusted and multivariable incidence rate ratios (RR) and 95% confidence intervals (CI) in relation to each outcome for both history of hypothyroidism and history of hyperthyroidism in the NHS and NHSII combined data. In addition, we evaluated EC and OC risk with the duration of having a history of each thyroid disease (<6 or ≥6 years, median time since diagnosis of hyperthyroidism and <8 or ≥8 years, median time since diagnosis of hypothyroidism), and we conducted tests for trend for duration of history of thyroid disease. In multivariable models, we adjusted for major lifestyle and reproductive factors for EC and OC determined *a priori* from the previous literature; the specific covariates and their specifications are provided in the footnotes to the main results tables. The covariates adjusted for in multivariable models represent cumulative histories as of the questionnaire immediately before the 2-year risk cycle (e.g., updated body mass index info was available every 2 years and at each biennial questionnaire cycle, we calculated the average of all of the available body mass index measurements and medical history (e.g., diabetes), and duration variables (pack-years of smoking) were updated at each cycle).

We performed several secondary analyses. To evaluate whether surveillance bias might explain potential associations, as the diagnosis of hypothyroidism and hyperthyroidism may be correlated with better access to health care/health consciousness that leads to greater frequency of ovarian/EC diagnoses, we conducted secondary analyses where we adjusted for high cholesterol (often an indication for thyroid testing) and having had a routine physical exam. Also, because hypothyroidism can be transient, to capture chronic conditions, we conducted alternative analyses where hypothyroidism was considered to be present only if it was reported at least twice during the follow-up period (and the date of diagnosis was the earliest reported date); because hyperthyroidism was rarely reported, we did not conduct similar analyses for hyperthyroidism as this had poor power. For EC, because body mass index is a strong risk factor, and body mass index also is strongly associated with thyroid status, we conducted analyses stratified by body mass index (<25 or ≥25 kg/m^2^, which is the median body mass index). For OC, we conducted analyses where we restricted analyses to OC with serous invasive/poorly differentiated histology.

## 3. Results

A history of hyperthyroidism was rare, with 0.6% of the total person-time of followup, while a history of hypothyroidism comprised 6–8% of the total person-time of followup in the cohorts (Table 1). In general, compared to those without any thyroid dysfunction, those with a history of hypothyroidism had higher body mass index, greater frequency of hypertension and diabetes, current estrogen use, tubal ligation, history of infertility, and family history of breast, ovarian, or uterine cancer. Compared to those without thyroid dysfunction, those with a history of hyperthyroidism had lower or similar body mass index, greater frequency of long-term smoking, hypertension (and diabetes in NHS), history of infertility, and family history of uterine cancer.

### 3.1. Endometrial Cancer

During 34 years of followup in the NHS, we documented 1131 cases of incident EC in women and during 22 years of followup in the NHSII, we documented 183 cases (Table 2). Age-adjusted and multivariable adjusted analyses were similar. Neither a history of hypothyroidism nor hyperthyroidism was significantly associated with risk of EC: the multivariable RR for a history of hypothyroidism was 0.91 (95% CI = 0.75, 1.10), while the RR for a history of hyperthyroidism was 1.43 (95% CI = 0.85, 2.39). The data suggested that a history of hypothyroidism, especially having such a history for 8+ years or greater (the median time), was nonsignificantly inversely associated with EC risk (RR of 8+ years with a history of hypothyroidism = 0.81; 95% CI = 0.63–1.04; *P*-trend with duration = 0.11). In contrast, having a history of hyperthyroidism for 6+ years (median time) was associated with a nonsignificantly increased risk (RR = 1.69; 95% CI = 0.86–3.30; *P*-trend with duration = 0.12). 

 In secondary analyses additionally controlling for report of routine physical exam and high cholesterol (an indication for thyroid testing), results were generally similar to the main results (Table 2). When we considered a history of hypothyroidism to be present only when it was reported at least twice during followup, we observed somewhat stronger inverse associations with a history of hypothyroidism (RR = 0.79, 95% CI = 0.62–1.00) and having had a history of hypothyroidism for 8+ years (RR = 0.78, 95% CI = 0.59–1.03; *P*-trend with time since diagnosis = 0.04). In analyses stratified by body mass index (<25 or ≥25 kg/m^2^), inverse associations with a history of hypothyroidism were mainly observed in those with higher than median body mass index: the RR for having a history of hypothyroidism for 8+ years was 0.77 (95% CI = 0.57–1.04; *P*-trend = 0.09) for those with body mass index ≥ 25 kg/m^2^, while the corresponding RR for those below the median body mass index of 25 kg/m^2^ was 0.96 (95% CI = 0.59–1.57; *P*-trend = 0.95); however, the p for interaction between body mass index and hypothyroidism in relation to EC was not significant (*p* for interaction was 0.15 for hypothyroidism status and 0.23 for duration of having a history of hypothyroidism).

### 3.2. Ovarian Cancer

During 34 years of followup in the NHS, we documented 908 cases of incident OC in women and during 22 years of followup in the NHSII, we documented 242 cases (Table 3). We observed that neither a history of hypothyroidism (RR = 0.84, 95% CI = 0.68–1.04) nor hyperthyroidism (RR = 0.67, 95% CI = 0.30–1.49) was significantly associated with OC. Having a history of hypothyroidism for 8+ years (median) was non-significantly inversely associated OC (RR = 0.87, 95% CI = 0.66–1.15; *P*-trend = 0.13). Having a history of hyperthyroidism for 6+ years (median) was not associated with OC (RR = 1.12; 95% CI = 0.46–2.72; *P*-trend = 0.95).

In secondary analyses where we additionally controlled for report of routine physical exam and high cholesterol, results were generally similar to the main results (Table 3). When we considered a history of hypothyroidism to be present only when it was reported at least twice during followup, we observed significant inverse associations between overall history of hypothyroidism (RR = 0.76, 95% CI = 0.58–0.99) and having had a history of hypothyroidism for 8+ years (RR = 0.81, 95% CI = 0.60–1.10; *P*-trend with time since diagnosis = 0.06). Analyses of OC with serous invasive histology or poorly differentiated histology showed similar weak nonsignificant associations with a history of hypothyroidism or hyperthyroidism as the main results (RR = 0.84, 95% CI = 0.64–1.11 for hypothyroidism and RR = 0.56, 95% CI = 0.18–1.76 for hyperthyroidism).

## 4. Discussion

In the largest cohort study to date to specifically evaluate the association between thyroid disorders and gynecologic cancer risk, we observed no significant associations of self-reported history of hypothyroidism and hyperthyroidism with either EC or OC risk. However, there was a suggestive weak inverse association of having a longer history of hypothyroidism with both gynecologic cancer outcomes and a modest positive association of having a longer history of hyperthyroidism with EC. 

Previous epidemiologic studies evaluating the impact of thyroid disorders on cancer risk have not been conclusive [20, 31–33]. Breast cancer is a hormone-dependent cancer and is one of the first malignancies to be associated with thyroid disease. Case-control studies in breast cancer patients observed a significantly reduced risk of breast cancer in women with untreated hypothyroidism [34]. Conversely, postmenopausal women with elevated thyroid hormone levels and subclinical hyperthyroidism had a higher rate of breast cancer than matched controls [35]. A recent meta-analysis of 28 studies evaluating any type of benign thyroid disorder or nodule and breast cancer risk reported no significant associations overall, but heterogeneity across studies limited the conclusions [36]. For total cancer risk assessment, Hercbergs et al. [27] conducted a large prospective study of almost 30,000 people and followed their thyrotropin levels (TSH) for over 9 years. Low levels, suggestive of subclinical hyperthyroidism, were associated with an increased risk of lung and prostate cancer but not breast or colon cancer. Despite our large cohort, we still had relatively small numbers of cancer cases with self-reported thyroid disease overall and did not observe a significant association for either type of thyroid disorder and EC in contrast to the findings of Brinton et al. [29]. 

 We observed a nonsignificant trend towards increased risk of EC with longer duration of history of hyperthyroidism (>6 years). The mechanism by which chronic hyperthyroidism might increase the risk of EC is not fully understood [37–40]. The endometrium is a complex system influenced by a delicate balance of steroid hormones receptors, growth factors, and cytokines. Women with hyperthyroidism have estrogen levels 2- to 3-fold higher compared to euthyroid women due to SHBG changes and decreased clearance of estradiol [17, 18]. Over time, chronic hyperthyroidism may indirectly increase serum estradiol levels and increase EC risk. Preclinical data has identified a class of cell surface receptors in the endometrium that bind thyroid hormone directly and expression is correlated with infertility and implantation defects [41–43]. Disordered growth of the endometrium is a precursor for endometrial hyperplasia and subsequent progression to carcinoma. Women with prolonged exposure to higher levels of thyroxine, as seen with hyperthyroidism, may be at risk for developing endometrial hyperplasia and EC by direct binding and proliferation, but further research is needed to investigate this association. In addition, the medical treatment of hyperthyroidism and the impact of thyroid blocking medications on the endometrium have not been well characterized. 

Conversely, we found a nonsignificant association between hypothyroidism and decreased EC risk. Previous studies have confirmed that hypothyroidism results in decreased estradiol levels and altered regulation from the HPA. The resulting anovulation and thin endometrial lining may potentially be protective over time and balance the obesity often associated with hypothyroidism [16]. The validation used in our cohort for self-reporting their diagnoses was excellent; however, confirming length of treatment and proper medication usage and, if possible, blood testing would be needed in future work. 

 We did not observe an association between history of hyperthyroidism or history of hypothyroidism and the risk of OC. Research on OC has been limited by the low incidence and inability to easily access the ovaries to understand the precursors to malignant transformation. The incessant ovulation theory links ovulation to epithelial damage, inflammation, and repair that can lead to OC and is a hormone dependent process. The ovaries express estrogen, progesterone, and thyroid receptors; however, their complex interactions are not fully understood and the impact on cancer development, if any, remains unclear [7, 44]. Our findings do not support a hormonal etiology as it relates to thyroid disease, although a modest association cannot be ruled out. Only two other reports have looked into this association with conflicting results. Brinton et al. [29] reviewed over 2500 OC cases from a Danish tumor registry and reported no association with thyroid disease; however, they were based on hospital diagnoses and only included 39 cases of thyroid disease. The other report was a population-based case-control study that suggested a significant relationship between hyperthyroidism and OC [45]. A follow-up study identified the ovarian surface epithelium as a target for thyroid hormone *in vitro* resulting in increased expression of estrogen [46]. Pooled or meta-analysis studies are needed to increase power to further examine these associations.

The strengths of this study include the fact that thyroid disease was ascertained before disease diagnoses, the large sample size of nearly 200,000 women, and the repeated and updated measures. A major limitation of our study was that we did not have measurements of thyroid hormone levels in our population and evaluated the associations with conditions related to having extreme levels of thyroid hormones, and thus we were not able to evaluate dose-response relations of thyroid hormones to risk of EC/OC. However, associations, if present, are likely to be detectable by evaluating chronic conditions that arise due to either abnormally very high or very low thyroid levels, and evaluations of the thyroid disorders was more feasible. Another major limitation was that we used self-reports of a history of hypothyroidism and a history of hyperthyroidism; while the validation studies of self-reports showed that a high percentage of all the self-reports were likely to be accurate in this population of nurses, there was likely underascertainment of thyroid disease in the population compared to if we had conducted in-person exams or measured thyroid hormone levels for all participants. This underascertainment would have likely caused some bias towards the null, which might explain some of the weak associations observed. We also lacked detailed information on the types, duration, and effectiveness of any treatments that participants might have received; therefore, if there were possible associations with a history of hypo- or hyperthyroidism, it is not clear whether it was the disease per se or treatments for these conditions that may be underlying the associations. Also, because hyperthyroidism was relatively rare in the population, there were few cases of cancer with a history of hyperthyroidism, and so we lacked power to detect modest associations. We also lacked power to conduct analyses by EC/OC histologic subtype of genetic predisposition. Because some thyroid dysfunction may be subclinical or mild in presentation, any associations with thyroid dysfunction may arise solely due to better medical surveillance. However, when we additionally adjusted for participant's history of receiving physical exams or a diagnosis of high cholesterol (which often prompts thyroid testing), results were similar, indicating minimal bias due to better surveillance of health outcomes in those with thyroid dysfunction. Finally, because this study was done in populations of mostly well educated, nonobese Caucasian women, there may be issues of generalizability ability to the general population; however, it is not likely that the biological mechanisms may differ by race, ethnicity, or socioeconomic status. 

In summary, a self-reported history of hypothyroidism or hyperthyroidism was not significantly associated with risk of EC or OC. However, these data were consistent with possible weak inverse associations with a history of hypothyroidism for both outcomes and adverse associations with a longer history of hyperthyroidism and EC risk. As research continues to evolve into the complex interactions between thyroid hormones, steroid receptors, and sex hormones on the female reproductive tract, we may identify new pathways associated with gynecologic cancer risk. Further studies are needed to clarify the origins of a possible relationship between abnormal thyroid hormone expression and EC risk and the potential for identifying new prevention or treatment strategies. 

## Figures and Tables

**Table 1:** Age and age-standardized characteristics of study participants by self-reported hypothyroidism and hyperthyroidism at the midpoints (1992 for Nurses' Health Study (NHS) and 1999 for NHSII) of the follow-up periods (NHS: 1976–2010 and NHSII: 1989–2011).

	NHS			NHSII		
	No thyroid dysfunction	Hypothyroidism	Hyperthyroidism	No thyroid dysfunction	Hypothyroidism	Hyperthyroidism
Percentage of person-time*	93.2	6.2	0.6	91.6	7.8	0.6
Number	76469	4534	388	88201	7634	622
Age, mean (SD), years	58.1 (7.2)	59.4 (7.0)	57.5 (7.1)	43.8 (4.6)	44.9 (4.6)	44.2 (4.6)
Body mass index, mean (SD), kg/m^2^	26.0 (5.0)	27.4 (5.6)	26.1 (4.8)	26.3 (6.1)	28.3 (7.0)	26.0 (6.0)
≥30 pack-years of cigarette smoking, %	19.4	16.2	26.8	2.6	2.5	4.8
History of hypertension, %	30.7	36.1	39.2	12.9	18.0	13.0
History of diabetes, %	5.0	7.5	6.6	2.0	4.6	2.3
Parity, mean (SD), number of children	2.9 (1.7)	2.9 (1.6)	2.9 (1.6)	1.8 (1.2)	1.8 (1.2)	1.8 (1.2)
Oral contraceptive use duration, mean (SD), years	2.0 (3.4)	2.0 (3.4)	2.2 (3.5)	4.7 (4.8)	4.4 (4.5)	4.7 (4.9)
Age at menarche, mean (SD), years	12.6 (1.4)	12.5 (1.5)	12.5 (1.3)	12.4 (1.4)	12.3 (1.5)	12.4 (1.5)
Current estrogen only postmenopausal hormone use (5+ years), %^†^	4.2	9.2	5.3	0.4	0.8	1.5
Age at menopause, mean (SD), years^‡^	49.7 (3.7)	49.7 (3.8)	49.6 (3.6)	46.3 (4.2)	46.0 (4.8)	46.1 (4.0)
Age at last birth, mean (SD), years^∣∣^	31.4 (4.4)	31.1 (4.2)	31.3 (4.4)	30.9 (4.7)	30.7 (4.8)	31.2 (4.7)
Tubal ligation, %	16.9	17.8	14.8	19.5	20.7	20.7
History of infertility, %	11.2	14.1	12.6	19.1	21.4	24.2
Family history of breast or ovarian cancer, %	12.7	13.2	12.4	10.1	10.5	10.1
Family history of uterine cancer, %	2.6	2.7	2.7	2.5	2.8	4.1

*Over entire followup (1976–2010 in NHS and 1989–2011 in NHSII).

^†^Among postmenopausal women.

^‡^Among women with natural menopause.

^∣∣^Among parous women.

**Table 2:** Hazard ratios (95% confidence intervals) of endometrial cancer by self-reported history of hypothyroidism and hyperthyroidism status in the combined Nurses' Health Study (NHS, 1976–2010) and Nurses' Health Study II (NHSII, 1989–2011).

Endometrial cancer	History of hypothyroidism			History of hyperthyroidism			
	No	Yes			No	Yes	
Number of cases	1176	123			1176	15		
Person-years	3,857,750	267,130			3,857,750	24,091		
Age-adjusted	1 (ref)	1.19 (0.98, 1.43)			1 (ref)	1.46 (0.87, 2.44)		
Multivariable-adjusted*	1 (ref)	0.91 (0.75, 1.10)			1 (ref)	1.43 (0.85, 2.39)		
Multivariable-adjusted^†^	1 (ref)	0.89 (0.74, 1.08)			1 (ref)	1.41 (0.84, 2.37)		
Multivariable-adjusted^‡^	1 (ref)	0.79 (0.62, 1.00)			—	—		
Multivariable-adjusted^∣∣^, BMI < 25 kg/m^2^	1 (ref)	1.08 (0.76, 1.55)			—	—		
Multivariable-adjusted^∣∣^, BMI ≥ 25 kg/m^2^	1 (ref)	0.87 (0.69, 1.09)			—	—		

	No	Yes <8 years^¶^	Yes 8+ years^¶^	*P* trend	No	Yes <6 years^¶^	Yes 6+ years^¶^	*P* trend

Number of cases	1176	56	67		1176	6	9	
Person-years	3,857,750	124,990	142,140		3,857,750	12,366	11,725	
Age-adjusted	1 (ref)	1.33 (1.02–1.75)	1.09 (0.85–1.40)	0.39	1 (ref)	1.18 (0.52–2.64)	1.74 (0.89–3.38)	0.09
Multivariable-adjusted*	1 (ref)	1.06 (0.81–1.40)	0.81 (0.63–1.04)	0.11	1 (ref)	1.16 (0.51–2.61)	1.69 (0.86–3.30)	0.12
Multivariable-adjusted^†^	1 (ref)	1.03 (0.78–1.35)	0.80 (0.62–1.03)	0.08	1 (ref)	1.14 (0.51–2.57)	1.68 (0.86–3.28)	0.12
Multivariable-adjusted^‡^	1 (ref)	0.82 (0.54–1.27)	0.78 (0.59–1.03)	0.04	—	—	—	—
Multivariable-adjusted^∣∣^, BMI < 25 kg/m^2^	1 (ref)	1.24 (0.76–2.03)	0.96 (0.59–1.57)	0.95	—	—	—	—
Multivariable-adjusted^∣∣^, BMI ≥ 25 kg/m^2^	1 (ref)	1.03 (0.74–1.43)	0.77 (0.57–1.04)	0.09	—	—	—	—

*Multivariable core model is stratified by age (in months), two-year cycle at risk, and cohort (NHS/NHSII) and is adjusted for body mass index (kg/m^2^; continuous linear variable and missing data indicator), cigarette smoking (continuous linear variable of pack-years of smoking and missing data indicator), family history of uterine cancer (yes/no), diabetes (yes/no), parity (continuous linear variable of number of children), oral contraceptive use history (never, <1, 1–4, 5–9, 10+ years of use, missing data indicator), age at last birth (30 or greater yes/no and missing data indicator), age at menarche (continuous linear variable and missing data indicator), age at menopause (continuous linear variable and missing data indicator), use of estrogen only postmenopausal hormone therapies (never user, past user with <5 years duration, past user with ≥5 years duration, current user with <5 years duration, current user with ≥5 years duration), use of estrogen plus progestin only postmenopausal hormone therapy (never user, past user with <5 years duration, past user with ≥ 5 years duration, current user with <5 years duration, current user with ≥5 years duration) and use of other postmenopausal hormone therapy (never user, past user with <5 years duration, past user with ≥5 years duration, current user with <5 years duration, current user with ≥5 years duration), and infertility (none, self is infertile, spouse is infertile, missing).

^†^To core model, additionally adjusted for greater health surveillance that may be associated with thyroid screening (report of physical exam and high cholesterol).

^‡^Hypothyroidism defined as being reported at least twice during followup; adjusted for the same variables as in core model. Not enough cases were available for analyses of the alternative definition for hyperthyroidism.

^∣∣^Stratified analyses by body mass index (BMI) categories defined by the median (25 kg/m^2^); BMI is a strong risk factor for both hypothyroidism and endometrial cancer. *P* for interaction with BMI was 0.15 for hypothyroidism status and 0.23 for duration of having a history of hypothyroidism.

^¶^Duration of having a history of hypothyroidism (median = 8 years) or hyperthyroidism (median = 6 years).

**Table 3:** Hazard ratios (95% confidence intervals) of ovarian cancer by self-reported history of hypothyroidism and hyperthyroidism status in the combined Nurses' Health Study (NHS, 1976–2010) and Nurses' Health Study II (NHSII, 1989–2011).

Ovarian cancer	History of hypothyroidism				History of hyperthyroidism			
	No	Yes			No	Yes		
*All epithelial /peritoneal cancer *								
Number of cases	1052	92			1052	6		
Person-years	4,427,881	329,761			4,427,881	28,582		
Age-adjusted	1 (ref.)	0.94 (0.75, 1.16)			1 (ref)	0.69 (0.31, 1.55)		
Multivariable-adjusted*	1 (ref.)	0.84 (0.68, 1.04)			1 (ref)	0.67 (0.30, 1.49)		
Multivariable-adjusted^†^	1 (ref.)	0.87 (0.70, 1.08)			1 (ref)	0.68 (0.30, 1.53)		
Multivariable-adjusted^‡^	1 (ref.)	0.76 (0.58, 0.99)			—	—		
*Serous histology only *								
No. of cases	615	56			615	3		
Multivariable-adjusted^∣∣^	1 (ref.)	0.84 (0.64, 1.11)			1 (ref)	0.56 (0.18, 1.76)		
	No	Yes <8 years^¶^	Yes 8+ years^¶^	*P*-trend	No	Yes <6 years^¶^	Yes 6+ years^¶^	*P*-trend

*All epithelial /peritoneal cancer *								
Number of cases	1052	36	56		1052	1	5	
Person-years	4,427,881	151,040	178,721		4,427,881	14,452	14,130	
Age-adjusted	1 (ref.)	0.88 (0.63–1.22)	0.98 (0.75–1.29)	0.55	1 (ref)	0.23 (0.03–1.63)	1.17 (0.48–2.82)	0.98
Multivariable-adjusted*	1 (ref.)	0.80 (0.57–1.11)	0.87 (0.66–1.15)	0.13	1 (ref)	0.22 (0.03–1.57)	1.12 (0.46–2.72)	0.95
Multivariable-adjusted^†^	1 (ref.)	0.83 (0.59–1.16)	0.90 (0.68–1.18)	0.21	1 (ref)	0.23 (0.03–1.62)	1.14 (0.47–2.76)	0.98
Multivariable-adjusted^‡^	1 (ref.)	0.63 (0.38–1.06)	0.81 (0.60–1.10)	0.06	—	—	—	—
*Serous histology only *								
No. of cases	615	23	33		615	1	2	
Multivariable-adjusted^∣∣^	1 (ref.)	0.85 (0.56–1.19)	0.84 (0.59–1.20)	0.17	1 (ref)	0.36 (0.05–2.53)	0.79 (0.20–3.20)	0.59

*Multivariable core model is stratified by age (in months), and two-year cycle at risk and cohort (NHS/NHSII) and is adjusted for body mass index (kg/m^2^; continuous linear variable and missing data indicator), family history of breast or ovarian cancer (yes/no), parity (continuous linear variable of number of children), oral contraceptive use history (never, <1, 1–4, 5–9, 10+ years of use, missing data indicator), age at menarche (continuous linear variable and missing data indicator), age at menopause (continuous linear variable and missing data indicator), use of estrogen only postmenopausal hormone therapy (never user, past user with <5 years duration, past user with ≥5 years duration, current user with <5 years duration, current user with ≥5 years duration), use of estrogen plus progestin only postmenopausal hormone therapies (never user, past user with <5 years duration, past user with ≥5 years duration, current user with <5 years duration, current user with ≥5 years duration), use of other postmenopausal hormone therapy (never user, past user with <5 years duration, past user with ≥5 years duration, current user with <5 years duration, current user with ≥5 years duration), hysterectomy (yes/no), tubal ligation (yes/no), and infertility (none, self is infertile, spouse is infertile, missing).

^†^To core model, additionally adjusted for greater health surveillance that may be associated with thyroid screening (report of physical exam and high cholesterol).

^‡^Hypothyroidism and hyperthyroidism defined only for those who reported the same diagnosis at least twice during followup; adjusted for the same variables as in core model.

^∣∣^Ovarian cancer with serous invasive histology (including papillary) or poorly differentiated histology; adjusted for the same variables as in core model.

^¶^Duration of having a history of hypothyroidism (median = 8 years) or hyperthyroidism (median = 6 years).
